# Debulking obstructing laryngeal cancers to avoid tracheotomy

**DOI:** 10.1016/j.bjorl.2019.07.004

**Published:** 2019-08-12

**Authors:** Fatih Gul, Yagmur Canan Teleke, Gokhan Yalciner, Mehmet Ali Babademez

**Affiliations:** a Yıldırım Beyazıt University School of Medicine, Department of Otorhinolaryngology, Head and Neck Surgery, Ankara, Turkey; b Ankara City Hospital, Department of Otorhinolaryngology, Head and Neck Surgery, Ankara, Turkey

**Keywords:** Airway obstruction, Laryngeal neoplasms, Debulking surgical procedures

## Abstract

**Introduction:**

Upper airway obstruction, secondary to neoplasms presenting with stridor, is traditionally treated by tracheostomy. However, this common procedure can potentially have an impact on the long-term outcome, with tumor implantation into the tracheostomized wound leading to peristomal recurrence after laryngectomy, with the risk of stomal recurrence.

**Objective:**

To describe our clinical experience with tumor debulking as an alternative treatment choice of tracheotomy in patients with advanced larynx cancer at a tertiary referral center.

**Methods:**

A retrospective chart review of 87 subjects who had advanced larynx cancer (T3/4) with airway obstruction from our institutional database was conducted. Medical records including demographics, daily notes during hospitalization, and operative notes were used for clinical data of patients. The strategy for maintaining the airway patency was tracheotomy (emergency or awake) and tumor debulking (laser or coblation). Endophytic and exophytic laryngeal tumors were also noted.

**Results:**

In 41/87 (47.1%) patients, a tracheotomy was performed as an initial treatment (11 were emergency, 30 were planned) to maintain airway patency. Tumor debulking was performed in 28 exophytic and 18 endophytic lesions by laser or coblation (17 and 29 patients, respectively). Tracheotomy was performed in 5 patients (4 endophytic, 1 exophytic) who could not tolerate debulking surgery due to aspiration, edema and dyspnea. Three of the them who required subsequent tracheotomy was in the laser group and two in the coblation group. The success rate of laser debulking was 82.35% (14/17) and 93.1% (27/29) for coblation.

**Conclusion:**

Tumor debulking is a safe and effective method to avoid awake tracheotomy in patients suffering from airway obstruction due to advanced larynx cancer.

## Introduction

Respiratory compromise from tumor obstruction can be a presenting symptom in patients with advanced aerodigestive malignancies. Upper airway obstruction, secondary to neoplasms presenting with stridor, is traditionally treated by tracheostomy. Aside from routine risks and morbidity, this common procedure can potentially have an impact on the long-term outcome, with tumor implantation into the tracheostomized wound leading to peristomal recurrence after laryngectomy, with the risk of stomal recurrence varying from 8% to 41%.^1^, ^2^, ^3^ In addition, malpositioned tracheotomy incisions may make salvage laryngectomies difficult. An emergent tracheostomy, with no prospect of reversal in the immediate future, can have a profound effect on the patient’s lifestyle and psychological well-being, at least in the initial period. An alternative procedure to provide the airway patency prior to the definitive treatment is partial excision of the obstructing tumor, called tumor debulking. Several surgical methods such as cold steel scalpel, forceps, cautery, or vaporization by the CO2 laser can be used for tumor debulking.

Coblation (plasma-mediated ablation), a type of high frequency electrosurgery, is an advanced technology that works via producing a plasma field using gentle radiofrequency energy and natural saline, resulting in low-temperature molecular disintegration of tissues.^4^ This technology has been used effectively in several ENT procedures such as tonsillectomy, adenoidectomy, reduction of hypertrophic nasal turbinate, snoring and sinus surgery. Despite widespread use of coblation, there is no study evaluating its overall safety and efficacy for management of debulking the tumor before definitive therapy in patients with upper airway obstruction secondary to malignancy.

In this study, we present our experience with tumor debulking using coblation technology to evaluate its safety and efficacy in patients with advanced laryngeal cancer presenting with respiratory compromise.

## Methods

### Study design

**Figure 1:**
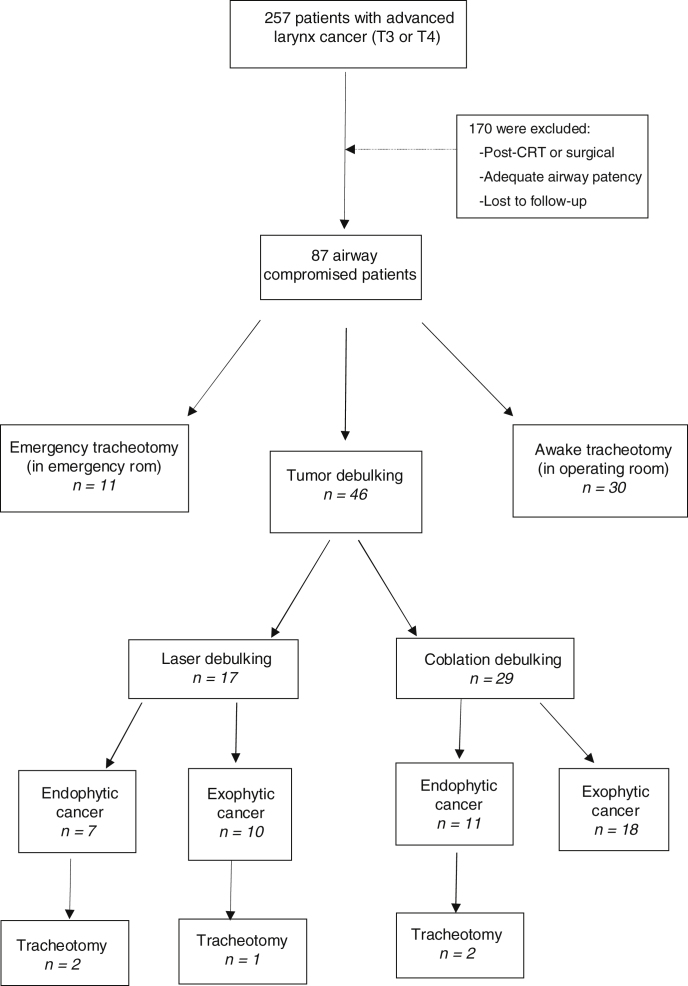
Schematic representation of retrospective analysis of patients.

This study was a single-center, retrospective chart review of subjects who had larynx cancer with airway obstruction from our institutional database. Our institutional review board approved this study. The database was used to retrieve the records of 257 patients with histopathological confirmed advanced larynx cancer between January 2012 and March 2019. Operative notes (tumor extent, width of rima glottis etc.) of patients with T3/4 supraglottic, glottic or trans-glottic cancers were reviewed to identify patients who underwent tracheotomy or tumor debulking due to dyspnea or stridor caused by airway obstruction. The choice of performing debulking or tracheotomy was determined according to the decision and experience of the surgeon. Emergency tracheotomy, awake tracheotomy, tumor debulking for endophytic and exophytic lesions were all initial treatment choices for airway- compromised patients with larynx cancer. Emergency tracheotomy was defined as cases in which airway patency of patients could not be achieved by endotracheal intubation in the emergency room. Awake tracheotomy was defined as patients whose airway was deemed too unstable to extubate or unable to intubate prior surgery in operating room. Laryngeal tumor volume obstructing the laryngeal passage was categorized as exophytic and endophytic lesions. Debulking of these tumor volumes were performed with laser or cold ablation techniques according to surgeon preference. Flow diagram of the patients is shown in Fig. 1. Medical records including demographics and daily notes during hospitalization were used for additional clinical data.

Excluding criteria were as follows:

Post-surgical or -radiotherapy patients with airway obstruction due to laryngeal cancer with or without edema;

Availability of adequate airway patency in patients with larynx cancer;

Patients lost to follow-up.

### Intubation and debulking of tumor

If intubation (2 attempts, each 2 min, facilitated by cricoid pressure) by endotracheal tube was unsuccessful, patients were shifted to the tracheotomy procedure. If successful intubation (mostly 5 or 6 size) was done, patients underwent debulking surgery plus direct laryngoscopy and endoscopic biopsy. A laser-resistance endotracheal tube was used in patients who underwent laser surgery, whereas no specified endotracheal tube was required for coblation technique.

Surgical removal of as much of tumor as possible in laryngeal airway was the main purpose of maintaining the airway patency. Based on this purpose, laser and coblation techniques were used for debulking tumor volume with an immediate effect for maintaining airway patency. The carbon dioxide (CO_2_) laser was used in all laser debulking cases to achieve clearance by using high-temperature vaporization for exophytic and endophytic tumor debulking. If hemorrhage was encountered, suction diathermy was used to control bleeding. The coblation was used in both exophytic and endophytic tumor volumes by using low-temperature radiofrequency and saline to create a plasma field that dissolves tumor tissue. Similar to chordotomy technique, debulking for endophytic tumors was performed 1–2 mm anterior to the vocal process, carrying laterally through the width of the tumor including the vocalis muscle on the obstructing side. Debulking was continued until an adequate airway was obtained. The procedure was considered successful if a tracheostomy was avoided until ultimate definitive treatment.

### Statistical analysis

SPSS vs. 22.0 was used for statistical assessment. Hospitalization time and time to definite treatment showed normal distribution in Kruskal–Wallis test and they were compared between tracheotomy and debulking groups by using Student *t* test. Statistical significance determined as *p* < 0.05.

## Results

A cohort of 257 patients was diagnosed as advanced larynx cancer (T3 and T4) at our institution. Eighty-seven of these suffering from stridor or airway obstruction caused by tumor obstruction were retrieved from the database. Of these, 11 presented with stridor requiring emergency tracheotomy in an emergency room (12.6%). Thirty patients underwent awake tracheotomy in our operating room (34.4%). Forty six patients underwent tumor debulking for maintaining airway patency to avoid tracheotomy (52.8%). No statistically significant difference was found between tracheotomy and debulking groups with regard to age and Body Mass Index (BMI) (*p* = 0.237 and *p* = 0.345, respectively). There were statistically significant differences between the two groups in terms of hospitalization time and time to definite treatment (*p* = 0.023 and *p* = 0.017, respectively). The clinical characteristics of the patients are listed in Table 1.

**Table 1:** Clinical characteristics of the laryngeal cancer patients with airway obstruction.

	Tracheotomy group (n = 41)	Tumor debulking group (n = 46)	*p*
Age (y)	43.2 ± 12.7	45.7 ± 14.3	0.237
BMI (kg/m^2^)	27.32 ± 3.1	26.4 ± 4.1	0.345
Sex (n)			
Female	10	12	‒
Male	31	34	‒
T stage (n)			
T3	12	19	‒
T4	29	27	‒
Hospitalization time (d)	24.3 ± 10.4	17.2 ± 8.5	0.023
Time to definite treatment (d)	16.2 ± 6.5	8.8 ± 7.1	0.017

BMI, Body Mass Index.

Twenty-eight exophytic tumor volumes underwent tumor debulking by using laser and coblation (10 for laser, 18 for coblation). Eighteen endophytic tumor volumes extending under the mucosa were reduced using coblation for 11 patients, laser for 7 patients. Among the patients who underwent tumor debulking, the exophytic ones were in T3 and T4, and the endophytic ones were in T4 classification. Five patients could not tolerate debulking surgery. 4/5 patients who underwent tracheotomy after debulking surgery had endophytic laryngeal cancer. Tracheotomy was performed in these patients due to aspiration and/or dyspnea. 1/5 patient with exophytic tumor, severe edema was developed on the first day and tracheotomy was performed. Time to subsequent tracheotomy was similar on both groups (1.6 day in laser, 1 day in coblation group). The success rate of laser debulking was 82.35% (14/17) and 93.1% (27/29) for coblation (Table 2).

**Table 2:** Details of the patients underwent laser and coblation tumor debulking.

	Laser debulking (n = 17)	Coblation debulking (n = 29)
Growth patterns (n)		
Endophytic	7	11
Exophytic	10	18
Subsequent Tracheotomy (n)	3	2
Time to subsequent tracheotomy (days)	1.6 (1.22)	1 (1.1)
Success rates (%)	82.35	93.1

## Discussion

In the early stage of laryngeal cancer, hoarseness is the most frequent symptom.^5^ However, respiratory compromise secondary to upper airway obstruction caused by tumor tissue can be an initial presenting symptom of exophytic or advanced glottic or transglottic tumors. There are three management options to provide a secure airway in these patients: emergency laryngectomy, tracheotomy or tumor debulking. To secure the airway and avoid tracheotomy, acoblator was used with increasing frequency for debulking the tumor in patients with advanced laryngeal tumors in our department. To our knowledge there is no study describing this procedure and its safety for tumor debulking. The purpose of our study is to describe our experience with this procedure, which we believe is a valid alternative to other debulking procedures and tracheotomy.

Tracheotomy remains the safest method of managing the upper airway compromised by tumor. However, emergency tracheotomy for obstructing laryngotracheal tumors increases the risk of stomal recurrence which has been cited to be from 8% to 41%.^3^ Stomal recurrence is associated with an approximately 90% mortality rate, and over 80% of the patients die in the first 24 months.^6^ Preoperative tracheotomy also creates a scarred field for future laryngectomy and results in a much more technically challenging procedure.

Tumor debulking, defined as a partial excision of the obstructing tumor, offers several advantages over emergency tracheotomy: the risk of stomal recurrence due to preoperative tracheotomy and requirement to placement of stoma from lower level is eliminated, the patient can be better evaluated in the preoperative setting and provide informed consent, the patient is often better prepared psychologically than after a tracheotomy. Thus, head and neck surgeons have performed tumor debulking for the past few years as an alternative to tracheotomy and emergency laryngectomy.

In a series of 109 patients with locoregionally advanced aerodigestive malignancies, 42 (38.5%) presented with airway obstruction. Of these, 28 (67.7%) underwent tracheotomy, and 11 (26%) underwent tumor debulking prior to definitive treatment. Of the 11 patients, 8 (82%) avoided a tracheotomy before, during, and after treatment.^7^ In our series, of the 87 patients suffering from airway obstruction caused by tumor obstruction, 46 (52.8%) underwent tumor debulking for maintaining airway patency and success rates were obtained as 82.35% and 93.1% for laser debulking and coblation debulking, respectively. Tumor debulking provided our patients up to 8 days of adequate airway control until definitive treatment was initiated. However, there was a significant delay (16 days) in beginning definitive treatment among patients who underwent tracheotomy as an initial emergency treatment. Our study also showed a significant prolonged hospitalization time in tracheotomy group. The requisite recovery period from tracheotomy accounts for a portion of this prolonged hospitalization time and delay in beginning of definitive treatment. However, the recovery from a debulking procedure is quick and does not lead to a delay in definitive treatment.

In our study, 5 patients required subsequent tracheotomy after earlier period of tumor debulking because of laryngeal edema. However, no patients needed tracheotomy after starting treatment, whereas in other series, a small percentage without initial airway symptoms did need tracheotomy due to chemoradiotherapy toxicities.^7^

For tumor debulking, several procedures have been used such as cautery, cold blade, laser or microdebrider and, in the literature there are a few studies evaluating the efficacy of diverse debulking procedures as an alternative method to tracheotomy in patients with upper airway compromised by tumor. Simoni et al. have described the use of the microdebrider to debulk the larynx tumor in their retrospective study and they reported a 96% immediate extubation rate.^8^ In the senior author’s experience, the microdebrider is much slower in removing the tumor, with more bleeding throughout the procedure, but is a credible alternative, particularly for the inexperienced laser surgeon. Vaughan et al., in the late 1970s, were the first authors to report, in 2 patients, the successful use of the C02 laser to debulk an obstructive endolaryngeal tumor prior to definitive therapy.^9^, ^10^ Since then, several studies have reported similar success rates for laser debulking. Davis et al., evaluated 10 carefully selected patients undergoing partial laser excision of laryngeal tumors and tracheotomy was avoided in nine patients with 90% of success rates.^11^ In another study, Paleri et al. evaluated the efficacy of the laser debulking procedure in avoiding a tracheotomy in 43 patients and they reported that tracheotomy was avoided in 91% of patients.^12^ Laccourreye et al. described 50 patients who underwent the procedure with the CO2 laser and the success rates were reported as 92.8% in patients in whom the C02 laser debulking procedure preceded definitive therapy with curative intent and 87.5% in patients in whom the C02 laser debulking procedure was part of palliative treatment.^13^ In accordance with the literature, we found the success rates of laser debulking as 82.3%. Three patients required subsequent tracheotomy throughout treatment. As our results support, laser debulking is an effective method to avoid tracheotomy, but in our opinion, it is often a slow and laborious procedure requiring proper equipment and a trained operating room staff. In addition, there is a risk of airway fire as a major complication.

As an alternative to laser debulking, in our department, we use the endolaryngeal coblation debulking for obstructing laryngotracheal tumors. In this study, 29 of 46 patients underwent coblation debulking and the success rate was found as 93.1%. For most patients, undergoing tumor debulking provided a safe airway prior to the definitive treatment period and, postoperative nasoendoscopic examinations revealed no reactive edema of the airway, for both laser and coblation techniques. However, 3 patients after laser debulking and 2 patients after coblation debulking required subsequent tracheotomy because of laryngeal edema. Although both methods have high success rates, coblation debulking seems to be a better alternative with more fast and uncomplicated applicability. The risk of intratracheal fire, a rare yet dreadful complication of laryngeal laser surgery, is also eliminated with the laryngeal coblation.

Our experience indicates that for the endophytic tumors, once the tumor is clinically shown to be T4 classification, the debulking method can be applied to include posterior cordotomy, different from the method used in exophytic tumors. In this way, successful results can be obtained by providing adequate airway patency in patients with endopyhtic tumors. In our series, 18 patients with endophytic tumors underwent tumor debulking with posterior cordotomy and adequate airway patency was provided in 14 of them, however, 4 patients required subsequent tracheotomy.

We recognized some limitations of our study, but despite these limitations we suggested that useful clinical conclusions could be drawn from our data. The first limitation was that our study was retrospective. The second, the decision for tracheotomy, laser or coblation debulking was determined at the time of dyspnea according the surgeon preference. Heterogeneity in surgical approaches weakened the power and focus of the study but on the other hand this heterogeneity might be considered a strong point for the fact that tumor debulking surgery could be an alternative treatment method to avoid early complications and morbidities of tracheotomy. The third limitation was that late complications of tracheotomy or debulking surgeries could not be observed because all the patients underwent a laryngeal surgery in a certain time. Therefore, the effect of tracheotomy and debulking surgeries on the recurrence of the disease or the surveillance of the patients could not be determined.

## Conclusion

This study shows that endolaryngeal coblation debulking of obstructing laryngeal tumors is a good alternative to other endolaryngeal debulking methods in preventing tracheotomy or emergency laryngectomy for obstructing laryngotracheal carcinomas. This technique is a safe and effective method with high success rates.

## Conflicts of interest

The authors declare no conflicts of interest.
